# Facilitating Consumers Choice of Healthier Foods: A Comparison of Different Front-of-Package Labelling Schemes Using Slovenian Food Supply Database

**DOI:** 10.3390/foods9040399

**Published:** 2020-03-31

**Authors:** Urška Pivk Kupirovič, Hristo Hristov, Maša Hribar, Živa Lavriša, Igor Pravst

**Affiliations:** 1 Nutrition Institute, Tržaška cesta 40, SI-1000 Ljubljana, Slovenia; urska.pivk.kupirovic@nutris.org (U.P.K.); hristo.hristov@nutris.org (H.H.); masa.hribar@nutris.org (M.H.); ziva.lavrisa@nutris.org (Ž.L.); 2 University of Ljubljana, Biotechnical Faculty, Jamnikarjeva 101, SI-1000 Ljubljana, Slovenia; 3 VIST – Higher School of Applied Sciences, Gerbičeva cesta 51A, SI-1000 Ljubljana, Slovenia

**Keywords:** front-of-package nutrition labelling schemes, health symbols, nutrient profiling, Nutri-Score, HSR, choices, Finnish heart, Keyhole symbol

## Abstract

Summary indicator front-of-package nutrition labelling schemes are gaining momentum. In Europe, an example of such a scheme is Nutri-Score, which was first introduced in France. Supported by additional research, the scheme has the potential to expand into other countries. Such a scenario opens a series of questions related to the use of Nutri-Score in the territories with pre-existing food labelling schemes. A key question is whether different nutrition labelling schemes would provide conflicting information for consumers when applied to same foods. The goal of our study was, therefore, to evaluate the alignment of different front-of-package nutrition labelling schemes. The study was conducted using cross-sectional data on the composition of selected categories of prepacked foods with high penetration nutrition/health claims and symbols in the Slovenian food supply. We evaluated a variety of existing front-of-package nutrition labelling schemes: three interpretive nutrition rating systems (Nutri-Score, Health Star Rating (HSR), Traffic light system), four health symbols (Protective Food symbol, Choices, Finnish heart, and Keyhole symbol), and also three nutrient profile models developed for other purposes (Office of Communications (United Kingdom, Ofcom), World Health Organization Regional office for Europe (WHOE) and Food Standards Australia New Zealand (FSANZ)). Overall, our results indicate that interpretive nutrition rating systems (i.e., Nutri-Score) are mostly less strict than the nutrient profiles of tested health symbols. A risk of conflicting information would happen in a scenario where food is eligible to carry a health symbol, but is at the same time rated to have lower nutritional quality by an accompanying interpretive nutrition rating system. When Protective Food symbol and Nutri-Score are used together, this would occur for 5% of foods in our sample. To avoid such risks, schemes for health symbols could be adapted to be stricter than interpretive nutrition rating systems used in the same territory/market, but such adaptations are challenging and should be well planned. While our study showed that, in most cases, Nutri-Score is a less strict model than tested health symbols, the rating-system approach might offer useful support and incentive for food producers towards gradual food reformulation.

## 1. Introduction

A recent comparison of the healthiness of packaged foods and beverages in different countries showed considerable variability in the nutritional quality of food around the Globe [1]. The finding that packaged foods and beverages are healthier in countries such as Australia and the UK indicates that well-planned policy approaches (including those related to food labelling) may have an important role in stimulating and supporting the food industry to reformulate products in the food supply and introduce healthier choices. The reformulation of processed foods is recognized as one of the key factors that could improve unhealthy diets and reduce the public health burden of noncommunicable diseases [2]. Producers of reformulated foods can follow one of two strategies when such foods are presented to consumers. One option is a “silent reformulation” [3], whereby small improvements in the nutritional composition are not exposed to the consumers, while another option is to highlight changes in the composition and present the product as a healthier alternative. The latter is very difficult due to regulatory requirements, because the use of comparative nutrition claims is only possible in cases where nutritional composition is changed considerably, likely resulting in products with very different sensory properties. Furthermore, binary labelling schemes (such as health symbols) are not sensitive enough to detect such reformulation changes. Therefore, a front-of-pack nutrition labelling scheme should be made in such a way that small steps in product reformulations are recognised to offer a strong incentive for producers to help them reformulate their products and systematically improve nutritional quality [4].

Information provided to consumers on food labels can affect consumers’ perceptions of foods on positive or negative way [5,6,7]. It is well established that foods labelled with health-related information can be more attractive to consumers, and nutrition labelling schemes (including health symbols) are considered a valuable element of labelling to guide consumers towards healthier food choices [8]. A variety of different (mostly voluntary) nutrition labelling schemes are being used on the front of the food packaging in different regions. While some were developed specifically to distinguish healthier foods (Keyhole provides an example of health symbol), some are more general and were developed to support consumers in interpreting the nutritional composition of foods (Nutri-Score provides an example of a summary indicator front-of-package nutrition labelling scheme) [8,9]. The appearance and message of the graphical element has an important impact on the consumer [10]. Considering the saturation of the media space and the plethora of brands that are competing to attract consumers’ attention, much effort is needed to achieve visibility and success with a labelling scheme [11], particularly when labelling of such schemes are not mandatory. As well as this, there is no international consensus about the superiority of one particular nutrient profile model, which could be used to rank the nutritional quality of foods [9]. This can be very confusing for consumers, because the same food product can be ranked as very healthy by one scheme, and not healthy by another scheme [12]. While nutritional labelling of the front-of-pack presents a very powerful tool to inform consumers, the information provided on food labels need to be trustworthy. To sustain/increase consumer trust in food labelling, it is critical to minimise confusion due to the use of different health-related information on food packages [12,13].

Nutrient profiling is clearly useful for some regulatory purposes, and research in this area is increasing [14]. The need for regulatory nutrient profiling was strongly highlighted for restricting the advertising of unhealthy foods to children [14], while other uses received less attention. For labelling purposes, different nutrient profiling models are used as the criteria for (mostly voluntarily) labelling foods with health symbols [8], while in Australia and New Zealand, nutrient profiling is also used to limit the use of health claims [15]. It should be mentioned that there is no scientific or other consensus on the superiority of one nutrient profiling (NP) system.

Front-of-package labelling (FOP) schemes need to be simple and give a quick comparison between products to help consumers to choose the healthier product in stores. More than 1 in 10 (11.4%) of the respondents in the UK study were assessed as limited in functional health literacy [16]. German eye-tracking study [17] found that the attention of the average consumer for the information on food labelling is on average only for 25–100 milliseconds. A study of eating habits in Slovenia [18] showed that 42% of Slovenian consumers never read food labels. Therefore, a front-of-package nutrition labelling scheme needs to draw attention and be comprehensive, to offer a quick comparison between products [19]. Egnell et al. investigated the understanding of five front-of-package nutrition labelling schemes (Health Star Rating (HSR), Multiple Traffic Lights, Nutri-Score, Reference Intakes, and Warning symbol) across 12 countries and showed that the Nutri-Score performed best in guiding participants to correctly rank nutritional quality of foods [20].

The use of front-of-package nutrition labelling schemes has a long tradition in Slovenia. A health symbol known as the Protective Food symbol was introduced in 1992 by the Society of Cardiovascular Health of Slovenia, aiming to help consumers make healthier food choices, and to support the reformulation of food products [21,22]. The introduction of Regulation (EC) 1924/2006 on nutrition and health claims made on foods influenced its use and the promotion of this scheme has stagnated a bit in the last decade. The symbol can be found on about 2% of prepacked foods in Slovenia [23], but its long-term presence on the market suggests that it is still very well recognised by consumers [22]. The criteria for the Protective Food symbol are based on a combination of food-traffic-light thresholds and regulatory criteria for specific nutrition and health claims. This is because the fact that this symbol is always presented together with a specific claim, providing the benefits of the food. It should be mentioned that several other health symbols are used in Europe, for example Sweden’s Keyhole symbol since 1989 [24], the Finnish Heart symbol since 2000 [25], and the Choices Programme symbol since 2006 [26]. On the other hand, summary indicator front-of-package nutrition labelling schemes are gaining momentum. In Europe, the successful launch of Nutri-Score in France [27] gained mostly positive responses from the scientific community [28] and World Health Organisation [29], although the response of the FoodDrinkEurope (European food industry association) was much less enthusiastic [30]. Nevertheless, considering the additional research behind this model [19,20], the Nutri-Score has the potential for expansion to other countries. Such a scenario opens a series of questions related to the use of Nutri-Score in territories with pre-existing food labelling schemes.

A key question is whether different nutrition labelling schemes would provide conflicting information for consumers when applied to the same foods. The goal of our study was therefore to evaluate the alignment of the different front-of-package nutrition labelling schemes using samples of prepacked foods available in the Slovenian food supply. The study was focused on a variety of existing front-of-package nutrition labelling schemes; we included three interpretive nutrition rating systems (Nutri-Score, HSR, Traffic light system), and four health symbols (Protective food, Choices, Finnish heart and Keyhole symbol). Additionally, comparison was made with three nutrient profile models, which are used in regulation to limit the use of health claims or the marketing of foods to children. This will allow the identification of major differences between models guide the improvement of existing nutrition labelling schemes, and drive evidence-based food policy decisions.

## 2. Materials and Methods

### 2.1. Food Database, Product Categorization and Nutritional Composition

The Slovenian branded foods dataset (CLAS, Composition and Labelling Information System) [31] was used for this analyses. In brief, the CLAS cross-sectional data on the composition of prepacked foods in the Slovenian food supply were collected during January–February 2015 in Ljubljana, Slovenia. To ensure the sample’s high representativeness, data were collected in grocery stores of retailers with accessible, nation-wide store networks and the largest market shares, comprising five shops (two mega markets, two supermarkets, and a discount market) of three major grocery chains (Mercator, Spar, and Hofer). Available foods were systematically photographed and recorded using web-based CLAS (Composition and Labelling Information System) software, developed by the Nutrition Institute (Ljubljana, Slovenia). Product photographs were used to collect product information, including data on nutrition composition. Product barcodes were used to avoid duplicates. Foods in the database were categorised using a classification system developed within the Global Food Monitoring Initiative (GFMI) [13]. For the purpose of this study, we included all foods with labelled nutrition composition information and sales data in the following five food categories, with notable prevalence of the Slovenian Protective Food symbol and/or nutrition/health claims (*N* = 1.370): Cereal and cereal products (breakfast cereals, pasta), dairy (cheese, yoghurt products), edible oils and emulsions (cooking oils). We should note minor deviations from GFMI food categorisation recommendations, due to specifics in the Slovenian food market. The modifications relevant for this study are in the pasta category, where we included pasta, noodles (pasta for soup, and not Asian noodles as a prepared meal, which is a limited product in the Slovenian market), and couscous.

### 2.2. Used Nutrient Profiling Models

The key characteristics of selected nutrient profiling models/scheme examined in this study are summarised in Table 1. The nutritional quality of foods was estimated with several front-of-package nutrition labelling schemes that are in use for FOP labelling in Europe: Keyhole [24,32], Dutch Choices symbol [33], Finnish Heart Symbol [34], Protective Food symbol [21], and Nutri-Score [28]. To gain a more complete picture, we also used interpretive nutrition ranking systems: Health Star Rating [35], the well-established FOP labelling system used in Australia; and the traffic light system that is also used in Slovenia (interpretation of nutrition declaration with smart phone application VešKajJeš [36,37]). Additionally, we used two nutrient profiles that are used to limit marketing to children, i.e., the Office of Communications (Ofcom, London, United Kingdom) nutrient profile model [38], which is used in the UK, and the World Health Organization Regional office for Europe (WHOE) nutrient profile model [39], that may be used by member states for the same purpose. As well as this, we included the Food Standards Australia New Zealand (FSANZ) nutrient profile criterion [15], which is used in the regulation to limit the use of health claims on foods in Australia and New Zealand.

Nutrient profiling for Keyhole, Protective Food symbol, Dutch Choices symbol, Finnish Heart Symbol, Nutri-Score, Health Star Rating, Ofcom, WHOE and FSANZ was conducted in line with previously refereed, publicly available profiling protocols (described in references provided in the previous paragraph). It should be noted that the Protective Food symbol uses the traffic light system (no red light) as a first step to evaluate nutrition quality, except in cooking oils and dairy products, where other criteria are used (cooking oils: eligible to carry authorised nutrition or health claims; dairy products: <3 g of fat per 100 g, <1.5 g of saturated fats per 100 g; [21]). In the case of food traffic light, the nutrient profiling was done using the previous version of the UK Food Standards Agency rules [40], which are still in use in Slovenia [36]. Profiling with food traffic light was also conducted for cooking oils, although these are single-ingredient products.

For each front-of-package nutrition labelling scheme, the proportion of “healthier” products was calculated. The following criteria were used for the different nutrient profiling systems: Keyhole, Protective Food symbol, Dutch Choices symbol, Finnish Heart Symbol: permitted to carry health symbol; Ofcom, WHOE: permitted for marketing to children; FSANZ: permitted to carry health claims; Nutri-Score: rated as dark green A or light green B (the same approach was used previously [13]); HSR: scored 3.5 or more (the same approach was used previously [12]); Traffic Light system: no red lights.

### 2.3. Data Processing and Statistical Analyses

Food product data were processed using computer programmes Microsoft SQL Server Management Studio 13.0, Microsoft Analysis Services Client Tools 13.0, Microsoft Data Access Components (MDAC) 10.0, Microsoft Excel 16.0 (Redmond, WA, USA) all Microsoft (Redmond, Washington, DC, USA), and programme tool Composition and Labelling Information System (CLAS) (Nutrition Institute, Ljubljana, Slovenia).

For each front-of-package nutrition labelling scheme, we calculated the proportion of »healthier products« available in the market per food category. We did not calculate percentage for overall categories or for parent categories, since the study was conducted only on selected food categories, and therefore our sample of foods is not representative of the overall market of branded foods. Sales weighted proportion of healthier products was also calculated based on each product’s 12 month sales data, which were provided by retailers, as described previously [31].

We investigated the agreement between Nutri-Score and other nutrient profile models with Cohen’s Kappa, as well as Protective Food symbol and other profile models. Agreement was determined using the Cohen’s Kappa ranges, as follows: 0.01–0.20 ‘slight’; 0.21–0.40 ‘fair’; 0.41–0.60 ‘moderate’; 0.61–0.80 ‘substantial’; 0.81–0.99 ‘near perfect’ agreement [41]. All analyses were performed using Data Analysis and Statistical Solution for Microsoft Excel (XLSTAT, Addinsoft, Paris, France).

## 3. Results

### 3.1. Proportion of “Healthier Products” by Tested Nutrient Profiling Models

**Figure 1:**
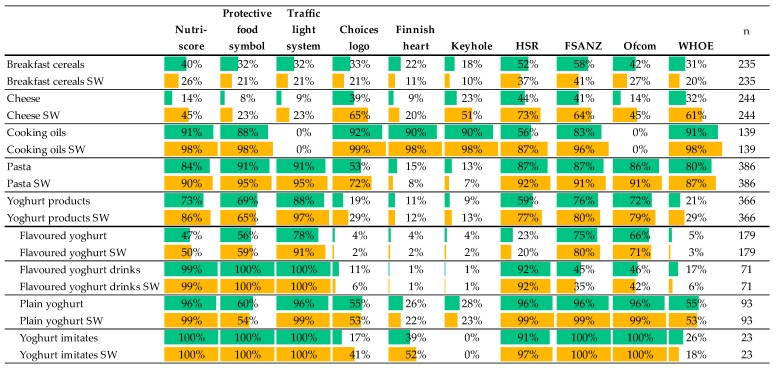
Proportion and sales-weighted (SW) proportion of product that are defined as »healthier foods« by different nutrient profiling models.

We have tested nutrient profile models on foods in the selected five food categories and observed differences in the proportion of foods they rank as healthier in each category (Figure 1). The food categories with the highest proportion of products eligible for A or B level by Nutri-Score were cooking oils (91%), pasta (84%), and yoghurts (73%) (Figure 1, green bars). The categories with the lowest proportion of products, eligible for A or B level by Nutri-Score, were cheese (14%) and breakfast cereals (40%) (Figure 1). Similar to Nutri-Score (91%) the highest proportion of healthier products by other FOP labelling schemes was also cooking oils, except for the traffic light system (0%) and HSR (56%). A lower proportion of healthier products than for Nutri-Score (84%) was also observed in pasta evaluated by the Choice symbol (53%), Finish heart (15%) and Keyhole (13%). A lower proportion of healthier products than for Nutri-Score (73%) was observed also for yogurt products with the Choice symbol (19%), Finish heart (11%) and Keyhole (9%). In breakfast cereals, except by HSR (52%), the proportion of healthier products was lower than by Nutri-Score (40%), with the lowest proportion found with Finish heart (22%) and Keyhole (18%). In cheese, surprisingly, the proportion of healthier products evaluated by the Choices symbol (39%) and Keyhole (23%) is higher than the proportion of healthier products evaluated by Nutri-Score (14%).

FSANZ and Ofcom, although used for different purpose then Nutri-Score, have a very similar proportion of healthier products to Nutri-Score, except in cooking oils (Ofcom 0%) and in breakfast cereals (FSANZ 58%) and cheese (FSANZ 41%). The food categories with the lowest proportion of products eligible for marketing to kids by WHOE were yoghurts (21%), breakfast cereals (31%), and cheese (32%).

Overall, a higher proportion of plain yoghurts is rated by all models as healthier compared to flavoured yoghurts and flavoured yoghurt drinks.

### 3.2. Sales Weighted Proportion of “Healthier Products” by Tested Nutrient Profiling Models

The sales-weighting approach was used to better estimate consumer choice over the availability of products in stores. Since the sales–weighting factors are the same across all scoring schemes, the comparison between nutrient profiling models indicates the proportion of healthier foods that consumers tend to buy. Figure 1 (yellow bars) shows the sales-weighted proportions of healthier foods increased in all food categories, indicating higher tendency of consumer to buy healthier products, by all tested nutrient profiling models, except for breakfast cereals (all), pasta (Finish hearth, Keyhole) and yogurt products (Protective Food symbol). A more detailed look in subcategory of yoghurt products shows that, in plain yogurt and flavoured yoghurts, the sales-weighted proportion of healthier products is decreased compared to the proportion of healthier products when evaluated by the Choices symbol, Finnish heart and Keyhole. A decrease in the sales-weighted proportion is also observed for flavoured yoghurt drinks, evaluated by the Choices symbol. For flavoured yoghurt drinks, the decrease in the sales-weighted proportion is also observed in FSANZ, Ofcom and WHOE nutrient profiling models. For plain yogurt and flavoured yoghurts, the sales weighted proportion of healthier products is also decreased compared to the proportion of healthier products when evaluated by WHOE.

### 3.3. The Agreement Between the Tested Nutrient Profiling Models

**Figure 2:**
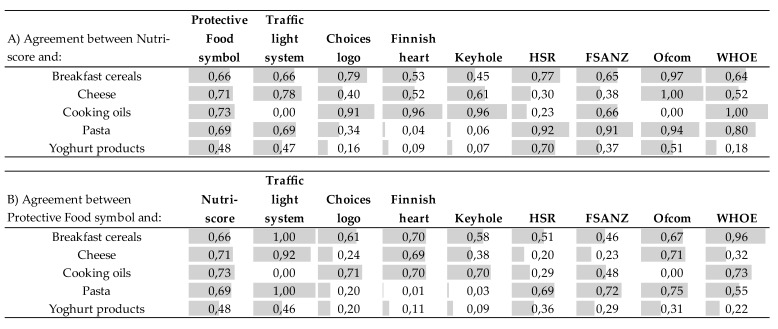
Agreement of (**a**) Nutri-Score or (**b**) Protective Food symbol with other nutrient-profiling models by Cohens kappa.

The agreement, based on Cohen’s Kappa, is near perfect between Nutri-Score and the Choices symbol (0.91), Finnish heart (0.96), Keyhole (0.96) for cooking oils, and HSR for pasta (0.92) (Figure 2a). A none-to-slight agreement is seen in cooking oils between Nutri-Score and traffic light (0). A none-to-slight agreement is seen in yoghurt products between Nutri-Score and Choices symbol (0.16), Finnish heart (0.09), and Keyhole (0.07), and in pasta between Nutri-Score and Finnish heart (0.04), Keyhole (0.06).

The agreement between Nutri-Score and Ofcom is near perfect for breakfast cereals (0.97), cheese (1.00) and pasta (0.94); between Nutri-Score and FSANZ (0.91) for pasta; between Nutri-Score and WHO for cooking oils (1.00). A none-to-slight agreement is seen in yoghurt products between Nutri-Score and WHOE (0.18).

The agreement between the Protective Food symbol (Figure 2b) and the traffic light system is near perfect for breakfast cereals, cheese and pasta, moderate for the yogurt products, and none for cooking oils. Agreement between the Protective Food symbol and Nutri-Score is substantial in all categories, except the yogurt products, where it is moderate.

### 3.4. Situation Scenarios of Food Labelling with the Use of a Health Symbol and Accompanying Interpretive Nutrition Rating System

**Figure 3:**
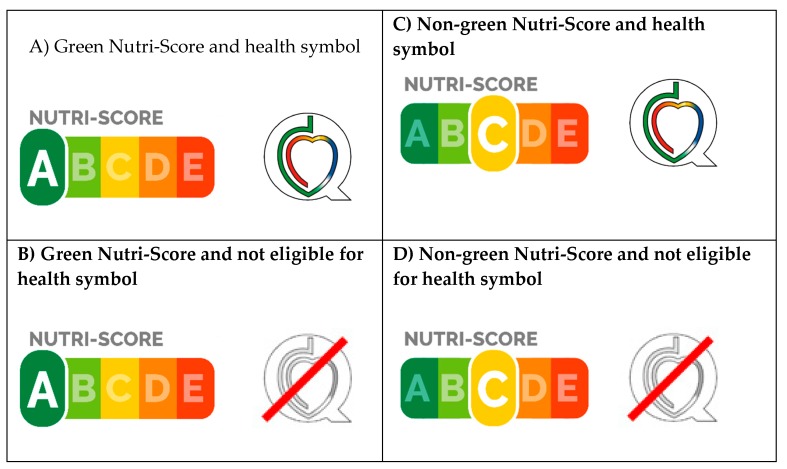
Possible real-life situation scenarios of food labelling with the use of a health symbol (example of Protective Food symbol) and accompanying interpretive nutrition rating system (example of Nutri-Score): (**A**) Green Nutri-Score and health symbol; (**B**) Green Nutri-Score and not eligible for health symbol; (**C**) Non-green Nutri-Score and health symbol; and (**D**) Non-green Nutri-Score and not eligible for health symbol.

We tested possible real-life situation of food labelling with the use of the health symbol and accompanying interpretive nutrition rating system. Test was done for a situation in which foods are subject to labeling with both Protective Food symbol (as an example of health claim) and Nutri-Score (as an example of nutrition labelling scheme). This is a realistic situation for Slovenian marketplace. Four possible scenarios, which would occur in such a situation, are presented in Figure 3. In our dataset, 5% of all sampled foods would be at same time eligible to carry Protective Food symbol, but labelled as non-green by Nutri-Score (Figure 3; Scenario C). In case of the Finish hearth symbol, such a Scenario would occur in 0.9% of sampled foods.

## 4. Discussion

The proportion of healthier products per food category, evaluated by Ofcom [42] and FSANZ [13], were consistent with previous studies. A good correlation of the Ofcom and Nutri-Score was also shown in our previous study for many food categories, except for cooking oils and yogurt products [13]. The difference between Ofcom and Nutri-Score in yogurt products is due to different categorisations of yogurt drinks, which fall into different categorisation groups (drink/food). That the alignment between HSR and FSANZ is problematic in categories of cooking oils and yoghurts was also reported previously [12].

From a public health perspective, the most concerning proportion of healthier products was in a category of breakfast cereals, where the proportion was below 50%, independently of the chosen nutrient profile for the evaluation—except for HSR (52%) and FSANZ (58%), where the proportion was still only slightly above 50% (Figure 1). Moreover, when considering sales data, the proportion of healthier products in breakfast cereals was reduced in all nutrient profiling models, indicating that market leading brands in breakfast cereals have a lower nutritional quality than other products present in the market. Breakfast cereals present an opportunity for producers to improve nutritional quality, as well as for policy makers to facilitate consumers’ choice of healthier products. A reverse effect is observed in cheese category, where it seems that market leaders have better nutrient profiles, since the sales-weighted proportion of healthier cheeses is increased over all nutrient profile models.

Even though Ofcom, FSANZ, HSR, and Nutri-Score have different purposes, results are showing that a very similar method is used to evaluate nutritional quality. These four models consider the same properties/nutrients for the evaluation of healthiness: both the elements to limit in a healthy diet (energy, saturated fat, total sugar, salt), as well as the beneficial constituents (content of fibre, protein and fruit/vegetable/nuts). These models differentiate between only a few food categories (two to six), which limits their sensitivity. Ofcom has only two categories: drinks and foods; FSANZ adds oils and fats; vile Nutri-Score adds cheese. Similarly, HSR differentiates between beverages, food, dairy beverage, dairy foods, cheese, oils and fats. A possible reason for observed differences in rating products as healthy could be in the differences in the assigned points for each nutrient and in the points used for the food category to rate the product as healthy. It is interesting that, even when differences in categorisation and method behind the model are small, like between Ofcom, FSANZ, HSR and Nutri-Score, the outcome might be very different [12]. Considerable differences in the evaluation are observed in cooking oils; some are related to an additional food category in the model (Ofcom vs. FSANZ), while others are related to a difference in the assignment of points (HSR vs. FSANZ). A simple categorisation system makes those nutrient profiling models easier to implement and use for the monitoring of the nutritional quality of products in the market. Having more categories considerably increase the complexity of the method of the nutrient profiling model [14]. While additional food categories in Nutri-Score might be needed for more sensitivity and the ability to differentiate foods into five rating grades [28], this topic was not in scope of our study.

Health symbols, as opposed to HSR and Nutri-Score, use a more complex food categorisation system (for example, Finish heart symbol uses 58 categories) and consider different selections of nutrients per category, with its own limit per nutrient. Keyhole, for example, also uses some additional criteria—like a minimum of 50% wholegrain flour for pasta. Tested health symbols might have more limited incentive effect for product reformulation, as they use a binary rating system (meeting or not meeting criteria for the symbol), only a few nutrients are included (fats, sugar, salt, etc.) and only one limit is set per food category. The WHOE scheme has consolidated 20 different food categories and set limits (depending on a food category) for energy, total fat, saturated fat, total sugar, salt, sweeteners and trans fats. WHOE considers only a limited number of nutrients and sets only one limit per nutrient in each food category [43].

All front-of-package nutrition labelling schemes included in study could facilitate consumers in making healthier choices, since they can follow nutritional guidelines and differentiate between healthy and less healthy products within a food category. In study, we assessed the performance of the Protective Food symbol, as a symbol with a long tradition to facilitate the choice of healthier products in Slovenia, and its consistency with health symbols used in Europe and other nutrient profile models. However, all the comparisons were performed with the Nutri-Score, a much more comprehensive model, which is gaining attention in many different European countries. In study we determined that Nutri-Score is mostly aligned with the criteria of the Protective Food symbol, indicating that Nutri-Score is a possible scheme that could be applied in Slovenia.

The reported study is important to inform future policy decision on the use of front-of-package nutrition labelling schemes as a possible tool for promoting healthy food choices, particularly in the European Union. Considering the well-established importance of healthy nutrition for the prevention of noncommunicable diseases [44], the reformulation of foods still presents a significant challenge, particularly when addressing food ingredients which are directly effecting the sensory properties of foods (sugar, fat, salt, etc.) [45]. To guide consumers’ food preference towards healthier choices, a complementary approach should also support the early development of healthy eating habits [46] and health literacy. However, for food producers to recognise reformulation efforts, rating scale schemes (such as Nutri-Score or HSR) might offer a higher incentive effect than existing binary schemes of health symbols. Potential benefit of rating scale schemes is also in more detailed information for consumers, but on the other hand, a drawback is that such schemes could be more complex to interpret, and that a consistency is needed with other schemes present on the same marketplace. Another issue that needs to be mentioned is that, in absence of mandatory use of health symbol labelling schemes, consumers cannot be sure if the specific food is not labelled with the symbol because it did not meet the criteria of the system, or if a supplier decided not to display the label for other reasons [47]. A possibly misleading approach that would also need to be avoided in case of rating scale schemes is selective use of the scheme only on those foods, which receive above-average rating. New technologies and sophisticated data collection can support implementation of nutrient profiling [47]. In Slovenia, for example, a smart phone application “VešKajJeš” was launched as part of the government funded “Innovative solutions for informed choices” programme [37]. This application enables users to check the nutritional quality of foods using food traffic light model. Application also use crowd-sourcing approach for collection of data on the composition of foods directly from users. We have evaluated the alignment of the different nutrient profiling models on the sample of prepacked foods available in the Slovenian food supply. Firstly, looking through the schemes, similarities are found in the selection of nutrients to limit (energy, saturated fat, sugar, salt) and nutrients to encourage (protein, fibers, fruits and vegetables). Although schemes are different in the number of product categories and limits set, they all divide products based on their nutritional quality. To help build on this consensus, our recommendation for the front-of-package nutrition labelling schemes as a possible tool for promoting healthy food choices is that they use a small number of categories, but still at least three groups of products, such as FSANZ (drinks, oils and fats, cheese and other foods). In contrast to setting limits (as in case in many health symbols), a cumulative scoring system allows some flexibility in product composition. For example, less favourable food constituents can be compensated with more favourable constituents, to still result in overall an improved nutritional composition.

Our main question was whether the introduction of supplementary interpretive nutrition rating systems, such as Nutri-Score, would present conflicting information to consumers when applied to foods labelled with existing health symbols, such as PF symbol. The results of our study show that, although Nutri-Score is in many cases less strict in comparison to tested health symbols, it is unlikely that this will result in conflicting information on the food label (Figure 3). In the (most likely) scenario of more strict health symbol criteria, foods eligible to carry health symbol would be also eligible for labelling with a green grade (A/B) Nutri-Score (Figure 3, Scenario A). However, if food pass the criteria for a green grade (A/B) Nutri-Score and not for the health symbol (Scenario B), this means that the food can be only labelled with Nutri-Score. Labels of such food cannot carry a health symbol, meaning that consumers will not be exposed to conflicting information. This is also the case in cases when food do not pass the criteria for a health symbol, and are graded as non-green by Nutri-Score (Scenario D). The only possibly conflicting scenario is Scenario C, where a specific food is eligible to carry a health claim, but is labelled as non-green by Nutri-Score. This can only happen in cases where the criteria for Nutri-Score are stricter than for health symbol. In our dataset, this was the case in up to 5% of foods. To avoid such scenarios, schemes for health symbols might be adapted to be stricter than supplementary nutrition labelling schemes, which could be used in the same territory/market, but such adaptations present considerable challenge and should be well planned and tested using wider range of food and beverage categories. For example, this study revealed that, in the case of the Protective Food symbol, the majority of conflicting cases were in yoghurts due to fat content, but other differences might be observed for food categories that were not investigated.

It should be noted that other studies also showed notable differences between the nutrient profiling models. For example, Labonté et al. [48] compared various models used for restricting the commercial marketing of foods and beverages of low nutritional quality to children, including WHOE and FSANZ and concluded that characteristics underlying nutrient profiling models should be carefully evaluated before the use for policy decisions.

A major strength of this study is in the variety of used nutrient profiling models and in the large dataset, which reflect the situation in the marketplace. Sales data enabled the sale-weighting approach, providing further insight into the food supply. On the other hand, our sample, at the same, time presents a limitation of the study. The database is representative only for the Slovenian food supply, but we should mention that Slovenia is part of the common European Union marketplace with the presence of all major international brands. A limitation of the study is also that we focused on specific food categories, and not the whole marketplace. We should mention that the whole dataset was extremely large and not manageable for assessment with such a variety of nutrient profiling models. Two approaches were possible, namely reducing the dataset using the randomisation approach (introducing sample selection uncertainty), or to focus it on specific food categories. We decided on the latter option, focusing on food categories with a notable penetration of nutrition/health claims/symbols. The representativeness of the sample could also be questioned, because the data was gathered in food stores in the capital (Ljubljana). However, we should note that data collection was conducted in nation-wide retailors, which provide over 50% of the national market in terms of volume.

## 5. Conclusions

In the study, we compared a variety of different nutrient profiling models, developed for various purposes. We have highlighted the differences between tested nutrient profiles for health symbols and other scoring systems. Our study showed that in rare situations consumers might be exposed to conflicting information on food labels. This could happen when a specific food is eligible to carry a health symbol but scored poorly in supplementary nutrition labelling schemes (for example, non-green by Nutri-Score). To avoid such scenarios, schemes for health symbols should be adapted to be stricter than those nutrition labelling schemes, which could be used in the same territory/market. With high obesity burden, the front-of-package nutrition labelling schemes might present an underexploited tool to help guide consumers choices towards healthier foods as well as encourage producers to offer more products with better nutrition quality. While our study showed that Nutri-Score (with A and B ratings) is a less strict model than health symbols that are used in Europe, a rating-system approach might offer useful support and incentive for food producers towards gradual food reformulation, but further studies are needed to address this topic.

## Figures and Tables

**Table 1:** Main properties for the used nutrient profiling models.

	Nutri-Score	Protective Food Symbol	Traffic Light System	Choices Symbol	Finnish Heart	Keyhole	HSR	FSANZ	Ofcom	WHOE
**Origin**	FR	SI	UK	NL	FI	SE	AU, NZ	AU, NZ	UK	EU MS for adaptation
**Year of issue**	2017	1992	2008	2007	2000	1989	2016	2016	2007	2015
**Use in other countries**	ES, BE	/	SI	NL, BE, CZ, NG	/	DK, NO, IS	/	/	/	/
**Purpose**	Food labelling: Rating system	Food labelling: Health symbol	Food labelling: Rating system	Food labelling: Health symbol	Food labelling: Health symbol	Food labelling: Health symbol	Food labelling: Rating system	Limit use of NH claims	Limit food marketing to children	Limit food marketing to children
**Considered as healthier where:**	Scores A and B	Allowed to carry symbol	No red light	Allowed to carry symbol	Allowed to carry symbol	Allowed to carry symbol	Greater than 3.5	Allowed to carry NH claim	Allowed for marketing to kids	Allowed for marketing to kids
**Visual**	Colour and letter-coded (from green to red, A-E) 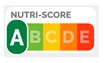	Symbol 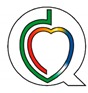	Colour coded (green, amber, red)	Symbol 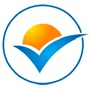	Symbol 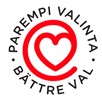	Symbol 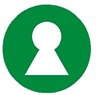	Numerical code (0,5-5 stars) 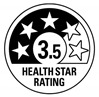	NA	NA	NA
**No. of food categories**	4	6	2	31	59	33	6	3	2	20
**Modeling method**	Score N-P points	Value within limits + NH	Value within limits	Value within limits	Value within limits	Value within limits	Score N-P points	Score N-P points	Score N-P points	Value within limits
**Nutrients included in the modeling method**										
**Energy**	Yes						Yes	Yes	Yes	For pasta only
**Total fat**		Yes	Yes		Yes	Yes				Yes
**Sat. fat**	Yes	Yes	Yes	Yes	Yes	Yes	Yes	Yes	Yes	Yes
**Total sugar**	Yes	Yes	Yes	Yes	Yes	Yes	Yes	Yes	Yes	Yes
**Salt**	Yes	Yes	Yes	Yes	Yes	Yes	Yes	Yes	Yes	Yes
**FVN**	Yes						Yes	Yes	Yes	
**Proteins**	Yes	For some					Yes	Yes	Yes	
**Fiber**	Yes	For some		Yes	Yes	Yes	Yes	Yes	Yes	
**Trans fat**				For some	For some	For some				For some
**Sweetener**						Yes				For some
**Added sugar**				For some	For some	For some				For some

N: negative points; P: positive points; NH: nutrition or health claim.
